# Numerical Analysis of Energy Dissipation and Frictional Effects in Aramid-Based Polymeric Fabrics Under Dynamic Loading

**DOI:** 10.3390/polym18020259

**Published:** 2026-01-18

**Authors:** Larisa Titire, Cristian Munteniță, Valentin Tiberiu Amorțilă

**Affiliations:** Faculty of Engineering, “Dunărea de Jos” University, 800008 Galati, Romania

**Keywords:** aramid fabric, polymeric composites, impact, friction coefficient, numerical simulation

## Abstract

Aramid-based polymeric fabrics are increasingly employed in lightweight protective and structural applications where high strength, flexibility, and impact resistance are required. Their response under high-velocity impact is governed by complex interactions among fiber properties, inter-yarn friction, and the mechanical behavior of the impacting body. In this work, three-dimensional finite element simulations were conducted in ANSYS Explicit Dynamics to investigate the coupled effects of the interfacial friction coefficient (μ = coefficient of friction = 0.0–0.5) and impactor material on the dynamic response of 24-layer plain-weave aramid panels. The numerical results reveal that low friction facilitates yarn mobility and localized penetration, whereas moderate friction enhances stress-wave dispersion and enables a more uniform activation of multiple fabric layers. At higher friction levels, penetration is further reduced, but localized stress concentrations may emerge due to constrained yarn movement. The constitutive properties of the impactor strongly influenced deformation modes and the efficiency of kinetic energy transfer to the composite structure. The simulated results are consistent with experimental data reported in the literature, confirming the predictive capability of the model. The study provides quantitative insight into the role of frictional interactions and impactor characteristics in optimizing the energy absorption and structural integrity of aramid-based polymeric fabrics subjected to high-velocity loading, contributing to the development of advanced lightweight protective materials.

## 1. Introduction

In many civil and industrial applications, high-velocity impact events represent a critical design and safety concern. Rotating components such as aircraft engine blades and high-speed railway axles operate in harsh environments, in which they may be struck by small foreign objects, including sand particles, metallic debris, hail, or birds. Such foreign-object impacts (FOD) can produce pits, cracks, surface deformation, and residual stresses, which may subsequently lead to fatigue-crack initiation and premature structural failure under high rotational speed and cyclic loading [1,2,3,4,5]. Similar mechanisms are observed in wind-turbine blades, where repeated exposure to lightning, sand erosion, rain, and ice accretion accelerates surface degradation and reduces service life [6]. Extensive experimental and numerical studies have therefore focused on the characterization of FOD-induced damage in metals, alloys, and composite structures, as well as on developing protection strategies such as surface coatings, composite reinforcements, and advanced inspection and monitoring techniques [6,7,8]. These investigations underline the broad relevance of high-velocity impact and foreign-object damage to the safety and reliability of rotating and load-bearing composite components in aerospace, railway, and wind energy systems.

Impact events in civil engineering contexts may arise from a wide variety of accidental scenarios, such as dropped tools, debris ejected from machinery, runway particles, or bird strikes. Understanding the behavior of materials under impulsive loading is therefore essential for predicting failure modes and designing safer lightweight structures. Impact is commonly defined as a collision between two or more bodies, in which the interaction may be elastic, plastic, fluid, or a combination of these responses. Among the governing parameters, impact velocity is regarded as one of the most influential quantities in impact dynamics [9].

A wide range of physical phenomena can occur after impact, including elastic, shock, and plastic wave propagation, fracture, fragmentation, perforation, and spallation [10]. In aircraft structures, impact damage may originate from multiple sources [11]. Generally, impact velocities are classified into four categories: low, high, ballistic, and hypervelocity [12,13,14,15].

- Low-velocity impact (<11 m/s) service-vehicle contact, dropped tools, cargo handling.
- High-velocity impact (>11 m/s) runway debris, propeller-ice shedding, hail, or bird strikes [7,16,17].
- Ballistic-range velocities (>500 m/s), small, high-speed debris or fragments generated by accidental explosions or machinery failures.
- Hypervelocity impact (>2000 m/s) impacts from micrometeoroids and orbital debris on spacecraft.

From a mechanical perspective, high-velocity impacts involving small-mass objects, such as debris, fragments, or dense particles, are especially critical because they can induce significant internal damage that may not be visible on the surface yet can severely reduce the residual strength of composite structures [18]. Consequently, an understanding of the dynamic response of polymeric composites under high-velocity loading conditions is essential, not only for safety assessments but also for the optimized design of next-generation lightweight structural and protective materials [19].

High-velocity impact events, commonly encountered in engineering and safety-related applications, involve the interaction of lightweight polymeric materials with rapidly moving objects or fragments. Such impacts can produce complex stress fields, localized deformation, and multiple damage mechanisms within the protective structure. In advanced protective systems, polymeric materials are often used in both ductile and rigid configurations to achieve a balance between flexibility, energy absorption, and structural integrity.

Soft protective systems are commonly designed by stacking multiple layers of high-performance polymeric fabrics. This multilayer arrangement ensures both flexibilities, allowing unrestricted body movement, and an enhanced capacity to dissipate energy during impact events. These systems are used in a range of lightweight protective and safety-related applications that require both comfort and reliable impact mitigation. When exposed to more demanding impact conditions, the protective performance can be further enhanced by the incorporation of rigid composite inserts, such as ceramic, polymer-matrix, or metallic plates—into the layered structure. Such hybrid configurations are also employed in environments where flexibility, resistance to repeated impacts, and mechanical adaptability are essential for maintaining protective efficiency [20,21,22].

Due to the numerous advantages offered by fiber-reinforced polymer composites, these materials are increasingly employed across a wide range of advanced structural and protective applications [18,23,24]. They combine high mechanical strength and stiffness with low density, providing lightweight solutions capable of withstanding high-velocity or repeated impact loading. Such composites contribute to reductions in overall weight, improvements in energy efficiency, enhanced mechanical reliability, and increased environmental sustainability. The evaluation of their performance is generally conducted using two complementary approaches, namely experimental testing and numerical simulation. A key aspect of impact-resistant composites is their capacity to dissipate kinetic energy while preventing complete material penetration during impact. This behavior is governed by the complex interaction between high-performance fibers and the polymeric matrix, in which energy is absorbed through mechanisms such as yarn stretching, matrix compression, delamination, and interfacial friction.

Aramid fibers, first developed in the late 1960s, rapidly gained widespread use in high-performance composite applications because of their outstanding strength-to-weight ratio and superior resistance to impact and deformation when compared with conventional polymer fibers such as nylon. These fibers are composed of rigid polymer chains interconnected through strong hydrogen bonds, which enable effective load transfer and efficient stress distribution. As a result, even polymer chains of relatively low molecular weight can exhibit remarkably high tensile strength and modulus [25,26,27,28,29].

Similarly to ultra-high-molecular-weight polyethylene (UHMWPE) fibers, aramid fibers exhibit a high degree of molecular orientation. They are characterized by high tensile strength, excellent impact resistance, good chemical and abrasion resistance, and low flammability [30]. Their density typically ranges from 1.44 to 1.46 g/cm^3^ [25,27,29].

The two-dimensional (2D) plain weave is the most fundamental and widely employed woven fabric architecture in impact-resistant composite applications. It is formed by interlacing warp yarns (oriented lengthwise) and weft yarns (oriented crosswise), resulting in a characteristic checkerboard pattern [31,32]. Woven fabrics have long been used in the development of flexible impact-resistant systems [33,34,35]. Improving the efficiency of such protective structures requires a detailed understanding of their high-velocity impact response, as well as the propagation of stress waves during loading [35,36].

In contrast to most existing meso-scale finite element studies, which typically assume a fixed impactor material model and limited or constant frictional conditions, the present work explicitly decouples and systematically analyzes the individual effects of interfacial friction and impactor constitutive behavior on the ballistic response of woven textile composites. By keeping the fabric architecture strictly constant and varying only the friction coefficient (μ = 0.0–0.5) and the impactor material model, the proposed approach allows a clear isolation of their respective contributions to stress-wave propagation, damage morphology, and impactor deceleration. This level of parametric separation is rarely addressed in previous meso-scale simulations and provides new mechanistic insight into how contact conditions and impactor response jointly govern energy dissipation pathways in multilayer woven fabrics. The numerical results are critically interpreted in comparison with experimental data and established literature, establishing a validated framework with direct relevance for the design and optimization of lightweight protective composite systems.

Ismail et al. [37] investigated the effect of pre-tension on the frictional behavior between single aramid fibers. A dedicated experimental setup was employed to demonstrate that elastic deformation—rather than tension-induced wrapping—governs the effective contact area between fibers.

Lincoln et al. [38] examined the influence of elastic properties on the frictional behavior of high polymeric materials. Using Hertzian contact theory, the deformation of surface asperities under load was analyzed, and this behavior was compared between metals and polymers. Measurements of the real contact area between a nylon hemisphere and a glass surface, together with friction tests on different nylon samples, indicated that polymers predominantly undergo elastic rather than plastic deformation. This elastic response was shown to contribute to the reduced friction observed for certain high polymers on rough surfaces.

Roselman and Tabor [39] investigated the friction and wear behavior of individual carbon fibers by rubbing them against a rotating cylindrical counter-surface composed of different materials. Friction forces were measured and interpreted using the capstan equation to relate the results to real contact area and interfacial adhesion. A comparison of different carbon fiber types revealed that friction strongly depends on fiber structure and counter-surface material. Additional wear experiments demonstrated the influence of environmental conditions and fiber type on dust formation, wear rates, and long-term fatigue behavior.

Chakladar et al. [40] investigate the influence of inter-tow angle and tow size on the frictional behavior of carbon fibers at the meso-scale. Their results showed that the angle between tows strongly affects tow–tow friction, particularly when the fibers are aligned, whereas tow size exerts only a minor influence. A finite element model was also developed to analyze inter-filament friction derived from low-level friction tests. The results demonstrated that filament-to-pulley friction significantly influences the load–displacement response, thereby identifying a range of intra-tow friction values at which inter-filament slippage initiates.

Gassara et al. [41] examined transverse friction occurring between fibers, a mode of interaction that has received limited attention in previous studies. To address this gap, a novel experimental device was developed to measure friction when two fibers cross obliquely, together with a mathematical model and a statistical method for estimating the friction coefficient with confidence intervals. The results indicated that transverse fiber–fiber friction is relatively stable and significantly lower than longitudinal friction, highlighting its importance for understanding the transverse mechanical behavior of yarns and multifilament strands.

Yanyan Chu [42] investigated the use of surface-modification techniques can increase inter-yarn friction in aramid and UHMWPE fabrics without adding weight. Through finite element simulations and experimental treatments such as APPCVD and sol–gel processes, it was demonstrated that friction can be effectively increased while preserving yarn tensile properties and overall fabric weight.

Feito et al. [43] studied the influence of impact angle on Kevlar fabrics under low-velocity impact conditions using a simplified numerical model. The objective was to develop a fast and computationally efficient tool for engineering applications, avoiding the complexity and high computational cost associated with mesoscale simulations. The proposed model was validated through comparison with experimental and numerical data available in the literature and was subsequently applied to analyze the effects of geometry, number of fabric layers, and impact angle on impact response. A mechanistic model and a surface diagram were also introduced to support the estimation of the critical impact velocity corresponding to complete energy absorption.

Huang et al. [44] numerically investigated the effects of inter-yarn friction and fabric architecture on low-velocity impact behavior. The results indicated that increased friction enhances energy transfer between yarns but may promote earlier localized failure. Among the investigated architectures, the plain weave exhibited the highest energy absorption capacity, whereas the 3/1 twill weave showed the lowest performance due to its more open configuration.

Bazhenov et al. [45] examined energy dissipation mechanisms in aramid fabrics with various weave patterns, with particular emphasis on the contribution of inter-yarn friction. The theoretical upper limit of the fibers’ ability to absorb energy under transverse impact was identified. The study showed that, in plain-weave fabrics, the maximum yarn pull-out force increases nonlinearly with the number of extracted yarns, whereas in twill weaves this relationship is linear. Consequently, altering the weave pattern represents an effective strategy for tailoring yarn pull-out forces. Due to inter-yarn slippage, the fabric exhibits a macroscopic response analogous to plastic material behavior.

Titire et al. [46] numerically analyzed the high-velocity impact behavior of layered aramid fabrics, evaluating 10- and 20-ply panels subjected to impact velocities of 398 m/s and 436 m/s. The results showed that the 20-layer configuration fully dissipated the incident energy at 398 m/s, whereas at 436 m/s partial perforation was observed. The study further highlighted that a broader distribution of longitudinal and transverse stress waves in thicker panels enhances energy dissipation and reduces the likelihood of localized failure.

Given the complexity of high-velocity impact interactions and the critical role of friction and constitutive material properties in governing energy dissipation pathways, a systematic numerical investigation of fabric–impactor impact scenarios is undertaken in the present work. By isolating the effects of friction coefficients and impactor material models, their influence on stress distribution, failure morphology, and the overall impact resistance of woven composite panels is clarified, thereby contributing to the rational design of next-generation lightweight protective composite materials.

## 2. Materials and Methods

The analyzed structure is composed of a 24-layer fabric, with each layer arranged in a 1/1 plain-weave configuration, corresponding to simple interlacing in which warp yarns pass alternately over and under weft yarns. This architecture ensures a uniform yarn distribution and promotes efficient force transmission along the principal material directions.

The cross-section of the yarns is modeled using a lenticular shape, which more accurately represents the geometry of fibers compressed during the weaving process. This cross-sectional profile is selected to achieve a realistic representation of yarn–yarn contact and the associated degree of material compaction. Consequently, the developed geometric model captures both the in-plane architecture of the woven fabric and the detailed yarn cross-sectional geometry, thereby enabling an accurate evaluation of the global mechanical response.

**Figure 1:**
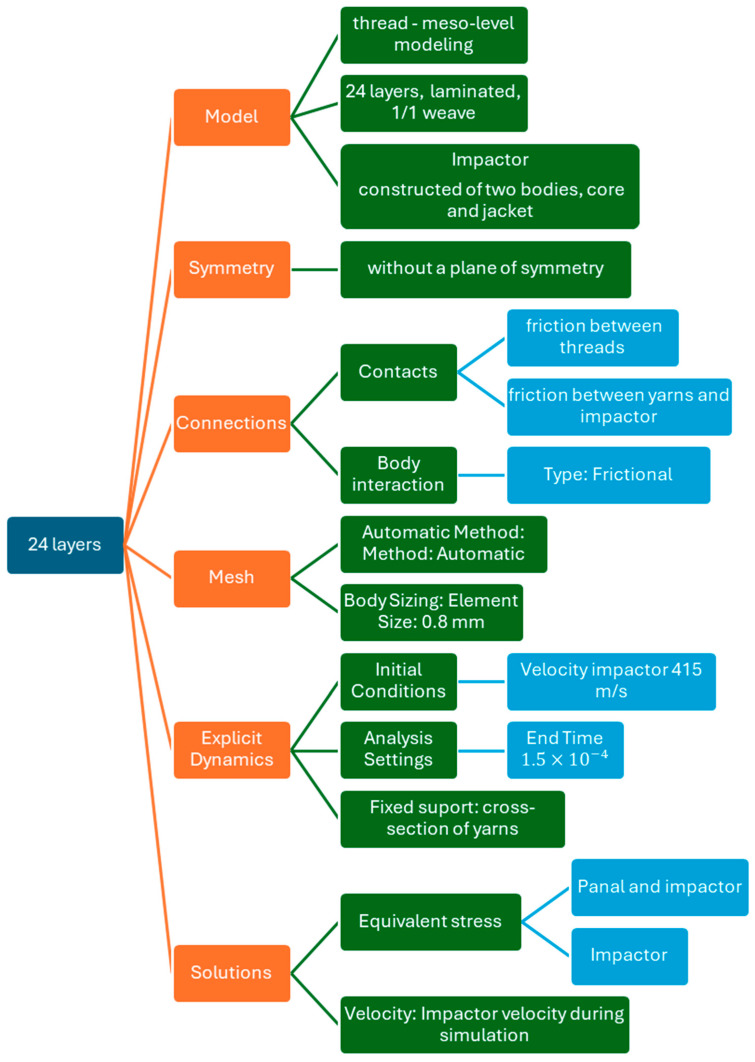
Modeling of Projectile–Fabric Interaction in 24-Layer Structure.

Numerical simulations were performed using the Explicit Dynamics module in ANSYS (https://www.ansys.com/, accessed on 1 September 2025) This approach enables precise modeling of the interaction between the impactor and the composite fabric, including frictional effects, plastic deformation, and the propagation of stress waves (Figure 1).

The importance of numerical simulations in high-velocity impact studies is well recognized. Such simulations allow substantial reductions in cost and time compared to experimental testing, which typically requires specialized facilities and expensive equipment. Moreover, numerical analyses provide detailed insights into stress distribution, strain-rate effects, and local yarn failure mechanisms, which are often difficult to obtain through experimental methods alone.

**Figure 2:**
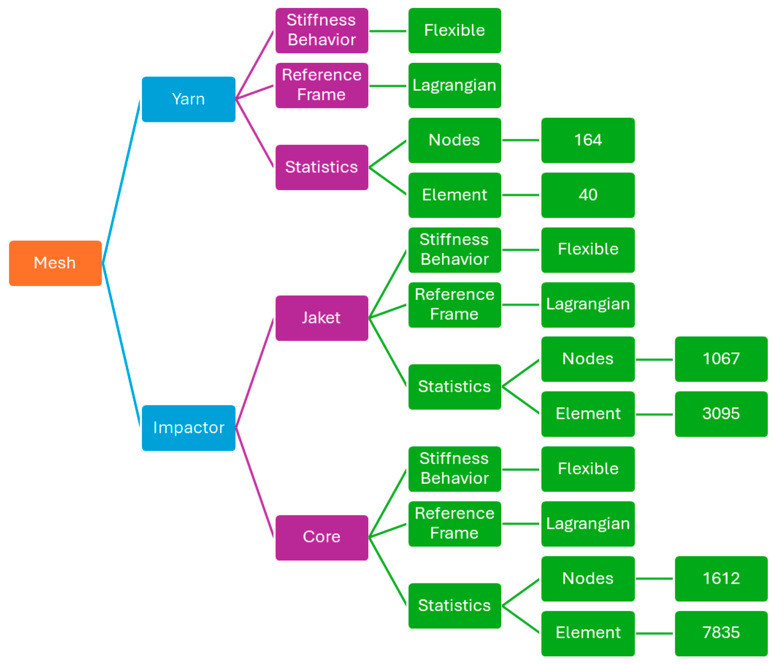
Mesh.

The numerical model was discretized using a fully Lagrangian formulation with flexible bodies for all components. The mesh was defined separately for the yarns, the impactor jacket, and the impactor core (Figure 2). The yarns were modeled as flexible Lagrangian bodies, discretized with beam/solid-type elements consistent with meso-scale yarn-level modeling. Each yarn cross-section was discretized using 40 finite elements, resulting in 164 nodes per yarn. This discretization ensures an adequate resolution of stress gradients across the cross-section yarn while maintaining numerical efficiency. The impactor jacket was modeled as a flexible Lagrangian body. Its mesh consists of 3095 finite elements and 1067 nodes, providing sufficient spatial resolution to capture local contact stresses and deformation during impact. The impactor core was also modeled as a flexible Lagrangian body. A finer discretization was adopted for the core, with 7835 finite elements and 1612 nodes, to accurately represent stress transmission and interaction with the textile structure during high-velocity impact. Across the full panel thickness, the fabric was modeled using 24 plies, with each ply discretized consistently using the same yarn-level mesh definition. This results in uniform through-thickness discretization and allows stress-wave propagation and inter-layer interactions to be resolved without introducing mesh-induced discontinuities.

The selected mesh represents a compromise between computational cost and numerical accuracy. Therefore, the presented results can be considered mesh-independent within the resolution required for meso-scale explicit dynamic simulations. No mass scaling or artificial mesh-based scaling techniques were employed. The explicit time step was governed by the smallest element dimension, ensuring numerical stability and avoiding dominance of the results by mesh or mass-scaling effects.

Because it enables the virtual testing of various material configurations, fabric topologies, and panel thickness prior to the fabrication of experimental prototypes, numerical simulation is therefore regarded as a crucial tool for optimizing protection structures. Furthermore, the modulus of elasticity, yield strength, density, or hardness of materials, as well as the coefficient of friction between yarns or between the projectile and the fabric layer, all have a significant impact on impact behavior.

Numerical simulation provides a controlled and cost-effective framework for analyzing these coupled processes across multiple scales (micro-meso–macro-scales). Meso-scale yarn-level models capture yarn bending, sliding, and rupture, and are particularly effective for investigating how inter-yarn friction and the constitutive behavior of the impactor influence load transfer and damage propagation [36,47]. Explicit dynamic finite-element solvers enable accurate resolution of the very short time scales and large deformations involved in high-velocity impact events. When combined with validated constitutive material models (e.g., Johnson–Cook for metallic components or elastic–plastic laws for polymeric and composite structures) and calibrated frictional contact definitions, such simulations can reproduce critical features observed experimentally and effectively guide material optimization prior to physical prototyping.

ANSYS Explicit Dynamics simulations allow for systematic evaluation of how variations in contact geometry, material constitutive parameters, and friction coefficients affect the overall impact response. As such, they represent an effective tool for the controlled and methodical design of composite systems aimed at maximizing energy absorption and minimizing material failure. In the present simulations performed using ANSYS Explicit Dynamics, no mass scaling was applied to the yarns, fabric layers, or impactor.

In the experimental campaign reported in [48], the 24-ply plain-weave panel was mounted on Roma Plastilina No. 11 backing material and secured using horizontal elastic straps, in accordance with the NIJ 0101.06 [49] testing protocol. Reproducing this complete mounting configuration at the mesoscale would require modeling the backing clay, straps, and a full-size panel, which is computationally prohibitive for explicit dynamic simulations at the yarn level.

Therefore, a reduced numerical model was adopted. The simulated fabric panel represents a local region of the textile, sufficiently large to capture the dominant impact and wave-propagation mechanisms, while remaining computationally tractable. The boundary conditions were defined by fully constraining the transverse faces of the yarns at the panel edges, effectively preventing rigid-body motion and approximating the confinement imposed by the backing and elastic straps in the experiment. No initial pretension was applied to the yarns, consistent with the experimental setup where the fabric was not pre-stressed before impact.

The impactor was assigned an initial velocity identical to that reported in the reference experiment used for validation. This velocity corresponds to the partial-penetration regime, where the projectile is arrested within the fabric thickness (experimentally at layer 16). Measurement uncertainty in the experimental velocity is reported in [48] to be within a few percent and was not explicitly modeled; instead, the nominal velocity value was used consistently across all simulations.

Although the numerical boundary conditions are simplified compared to the experimental fixture, the adopted approach preserves the local stress distribution, yarn failure mechanisms, and penetration/arrest behavior in the impact zone. This is confirmed by the good qualitative agreement between numerical predictions and experimental observations in terms of stopping depth, damage morphology, and impactor deformation.

To evaluate the influence of the impactor’s constitutive properties and the friction coefficient on the impact response, two variants characterized by different impactor material parameters and several scenarios with varying friction coefficients—both between the impactor and the yarns, and between adjacent yarns—are analyzed (Table 1).

Eight friction cases are analyzed, corresponding to friction coefficients μ = 0, 0.10, 0.20, 0.30, 0.40, 0.23, and 0.18 applied to both yarn–yarn and projectile–yarn contacts.

The material properties of the yarn (Table 2) [28,29,50,51,52,53,54,55], are identical in both numerical simulation scenarios. In simulation variant 1, the impactor is modeled as an assembly with two components: the jacket and the core. The jacket is described by an elastic-plastic model with bilinear hardening, and the core is defined by the Johnson-Cook constitutive law. The detailed properties are presented in Table 3 (jacket) and Table 4 (core).

In simulation scenario 2, the impactor is also modeled as an assembly consisting of a jacket and a core. The jacket is described using an extended Johnson–Cook constitutive model with a shock equation of state (Shock EOS Linear), which enables the representation of rate- and temperature-dependent plastic behavior, as well as the associated phenomena of compressibility at high impact velocities. The core is defined, as in the simulation scenario 1, by the Johnson–Cook constitutive law, accounting for both strain rate and temperature effects. The detailed material properties are presented in Table 4 (core) and Table 5 (jacket).

The material properties of the impactor are selected with the help of the literature, [50,56,57,58,59,60,61,62].

In all simulations, the geometry of the yarns and the impactor, the boundary conditions, the initial conditions (impact velocity), and the discretization parameters are kept identical. Contacts are defined as frictional in ANSYS Explicit Dynamics.

Aramid yarns are modeled as homogenized, continuous solid entities at the meso-scale. This assumption is commonly adopted in meso-scale finite element models of woven fabrics, where the objective of maintaining reasonable computational efficiency is to capture global load transfer, stress-wave propagation, and yarn-level failure mechanisms.

The mechanical properties assigned to the aramid yarns are consistent with values reported in the literature, with tensile strengths in the range of 2–3 GPa and elastic moduli between 70 and 120 GPa, representative of high-performance aramid fibers used in impact-resistant textile composites.

Although aramid fibers may exhibit some degree of strain-rate sensitivity, especially under high-rate loading, previous studies show that their elastic modulus is only weakly affected by strain rate within the range relevant to ballistic and high-velocity impact applications. Therefore, strain-rate effects are neglected in the present study, and a rate-independent elastic–brittle material model is considered sufficient to describe the dominant impact response mechanisms at the yarn scale.

This modeling strategy enables a consistent comparison between different impact scenarios while isolating the effects of the friction coefficient and impactor material properties on stress distribution, damage evolution, and energy absorption in the woven composite panel.

The metallic components of the impactor (jacket and core) are modeled using the Johnson–Cook (J–C) plasticity model. The J–C framework follows the original formulation proposed by Johnson and Cook and is widely adopted for high-strain-rate deformation and impact simulations of metals.

The J–C parameter sets used for the jacket/core are selected from published experimental characterizations reported for bullet materials (e.g., lead core and brass/copper jacket) tested over relevant strain-rate regimes. High-strain-rate data and constitutive identification for lead core and brass jacket materials have been reported in the literature and provide a consistent basis for the parameters used in the present work.

For the extended J–C + EOS variant, a Mie–Grüneisen-type equation of state (EOS) is employed to account for shock-compression effects at high loading rates. The EOS constants are chosen according to commonly adopted tabulated values for copper/brass-type alloys (jacket) and lead (core) used in impact dynamics literature and hydrocode material libraries, ensuring physically reasonable wave speeds and volumetric response under compression. Energy balance checks and comparison against the experimental deformation mode (mushrooming/petaling) are used as validation indicators for the selected parameterization.

In the present numerical model, failure of the aramid yarns is governed by a plastic strain–based failure criterion, defined through a critical equivalent plastic strain value, denoted as EPS = 0.04. Once this threshold is exceeded locally, the material is considered to have lost its load-carrying capacity. Failure is implemented using an element erosion (element deletion) criterion. Specifically, when the equivalent plastic strain in an element reaches the prescribed EPS value, the element is removed from the computational domain. This erosion criterion is used to represent local yarn rupture and fragmentation observed experimentally under high-velocity impact loading.

The chosen EPS value (0.04) is consistent with reported failure strains for aramid yarns under tensile and dynamic loading conditions, as documented in the literature. Experimental studies report ultimate tensile strains typically in the range of 2–4% for aramid fibers, depending on yarn construction, strain rate, and testing configuration. Accordingly, the selected value represents a physically realistic onset of yarn rupture at the mesoscale level.

In the present study, contact interactions are modeled using the Automatic Frictional Contact formulation available in ANSYS Explicit Dynamics. This contact type activates frictional sliding contact between any exterior node and any exterior face of the interacting bodies and automatically detects and tracks individual contact events throughout the simulation. The formulation is symmetric, meaning that nodes from each body can alternately act as contact nodes or target faces during the impact process [63].

A Coulomb friction model is adopted to describe tangential contact behavior. Friction is activated by prescribing a friction coefficient (μ) different from zero, so that the friction force is proportional to the normal contact force (F = μR). In accordance with the ANSYS Explicit Dynamics formulation, the friction coefficient can be defined as either constant or velocity-dependent. In this paper, a constant friction coefficient is used for all scenarios, unless otherwise explicitly specified [63].

For scenarios S1–S14, the same friction coefficient is applied consistently to both yarn–yarn and yarn–impactor contact pairs to isolate the effects of impactor material properties and failure mechanisms. In scenario S15, different friction coefficients are assigned to yarn–yarn and yarn–impactor interactions to explicitly investigate their individual influence on stress distribution, energy dissipation, and stopping capability. This distinction is now clarified to avoid ambiguity.

Tangential damping is implicitly included through the penalty-based contact algorithm and frictional sliding formulation. No additional numerical damping is introduced. Energy dissipation through frictional sliding is continuously tracked via the contact energy output, allowing verification that frictional work contributes physically to the total absorbed energy during impact.

The selected friction range (μ = 0.0–0.5) is consistent with values reported in the literature for aramid yarns and woven fabrics under dry contact conditions [38,42,64,65,66]. Lower values correspond to smooth or untreated yarn surfaces, while higher values represent coated, surface-modified, or tightly interlocked fabrics. In the absence of direct experimental friction measurements for the specific fabric considered, a parametric sweep was adopted to bracket realistic contact conditions and evaluate their influence on stress redistribution, damage morphology, and energy absorption.

## 3. Results

While longitudinal stress waves are propagated away from the impact point along the axes of the principal yarns at approximately the speed of sound, the impacting body induces transverse deflection in the primary yarns during contact. The process of energy absorption in the fabric is highly complex due to the large number of warp and weft yarns that may be engaged by the impactor. This complexity arises from the fact that the impactor may strike yarn interlacements, individual yarns, or the spaces between them. The resulting mechanical response becomes particularly intricate when the impact event involves only a limited number of yarns within the affected region [34,67].

During high-velocity impact on a composite target, the kinetic energy of the im-pactor is dissipated through a variety of damage and energy-absorption mechanisms [18]. These include localized material compression at and around the impact point, cone-shaped deformation on the rear surface, tensile loading of primary and secondary yarns, matrix cracking and delamination, shear plugging, and frictional interactions between the impactor and the target [18,34]. Each of these mechanisms contributes to the overall energy dissipation process and influences the deceleration or arrest of the impactor, depending on the impact severity and the structural configuration of the target. Understanding these phenomena is essential for evaluating the dynamic behavior of layered composite materials subjected to high-velocity impact.

When a textile composite panel is subjected to impact, the incident kinetic energy is distributed among multiple mechanisms: compression of the layers directly beneath the impactor, propagation of transverse and longitudinal stress waves, yarn stretching and rupture, shear plugging, yarn pull-out, delamination, and frictional work at yarn–yarn and yarn–impactor interfaces [68]. The relative contribution of each mechanism strongly depends on the impact velocity, the material response of the impactor, and the structural characteristics of the fabric—such as weave pattern, areal density, and number of plies—as well as interfacial properties like the coefficient of friction. Consequently, panels with identical textile architectures may exhibit markedly different impact responses depending on the constitutive behavior of the impactor and the contact conditions.

**Figure 3:**
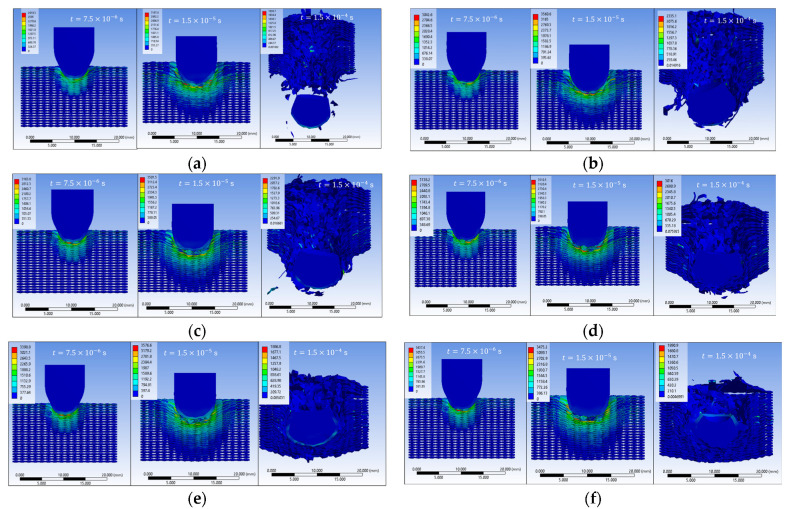

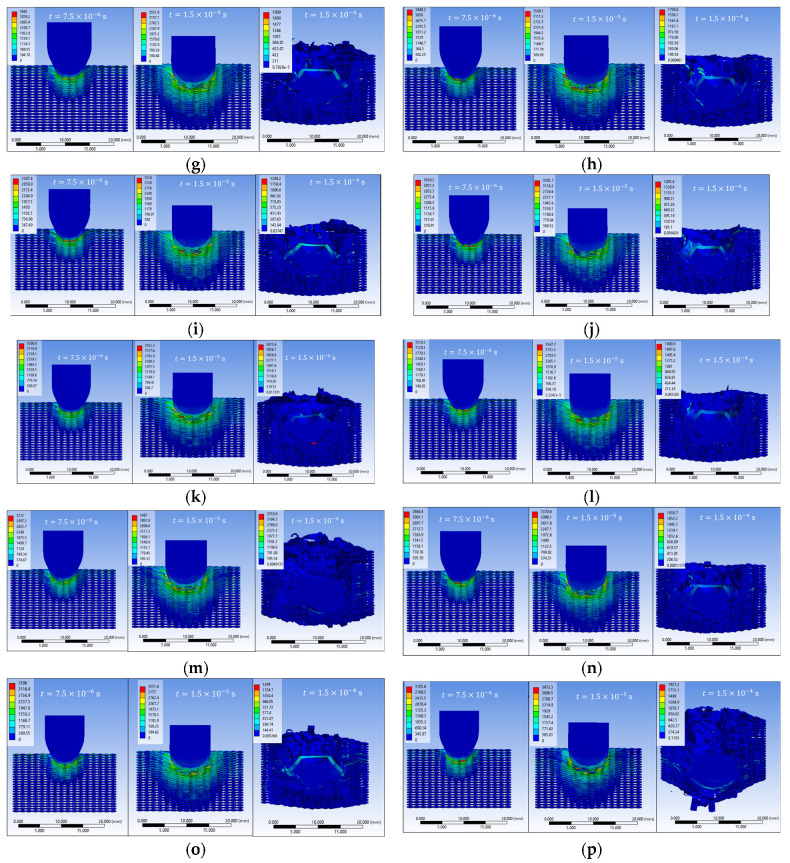
Distribution of equivalent stress (von Mises) in the yarns at different time steps during impactor penetration; (**a**) S1; (**b**) S2; (**c**) S3; (**d**) S4; (**e**) S5; (**f**) S6; (**g**) S7; (**h**) S8; (**i**) S9; (**j**) S10; (**k**) S11; (**l**) S12; (**m**) S13; (**n**) S14; (**o**) S15; (**p**) S16.

The results of the sixteen numerical simulations (S1–S16) provide a comprehensive view of the impact behavior of the textile panel under varying contact conditions, friction coefficients, and impactor constitutive variants (Figure 3). Despite these parametric differences, the simulations reveal a set of consistent response patterns that govern stress evolution, damage initiation, and penetration outcome.

At the initial impact stage (t = 7.5 × 10^−6^ s), all simulations show highly localized von Mises stress concentrations in the contact area between the impactor and the upper fabric layers, accompanied by the propagation of transverse and, in some cases, longitudinal stress waves. The maximum stress values obtained across the sixteen cases are in the range of approximately 2900–3500 MPa, with the main wires carrying most of the initial load, while the secondary wires remain weakly stressed.

During the intermediate stage (t = 1.5 × 10^−5^ s), the numerical results consistently indicate stress levels exceeding the wire strength, leading to wire rupture, pronounced compression of the upper layers, and an expansion of the stress field across multiple layers. Several simulations exhibit asymmetric stress distributions, reflecting the influence of fabric anisotropy, frictional contact, and impactor deformation. This stage marks the transition from localized damage to a more global involvement of the panel in impact energy dissipation.

In the final stage (t = 1.5 × 10^−4^ s), the sixteen simulations separate into two distinct response regimes. The first group of scenarios evolves toward complete perforation, characterized by the successive failure of all layers and, in some cases, the exit of the impactor from the panel. A second group results in partial penetration or impactor arrest, where damage remains localized in the impact region, and several lower layers preserve their structural integrity. In these cases, the impact energy is dissipated through combined mechanisms of compression, bending, tension, and local wire rupture.

Overall, the results of the sixteen simulations demonstrate that the penetration outcome and damage morphology are controlled by the interaction between stress distribution, wire orientation, frictional effects, and impactor material behavior, confirming the dominant role of the main wires and contact conditions in the ballistic response of the textile structure.

**Figure 4:**
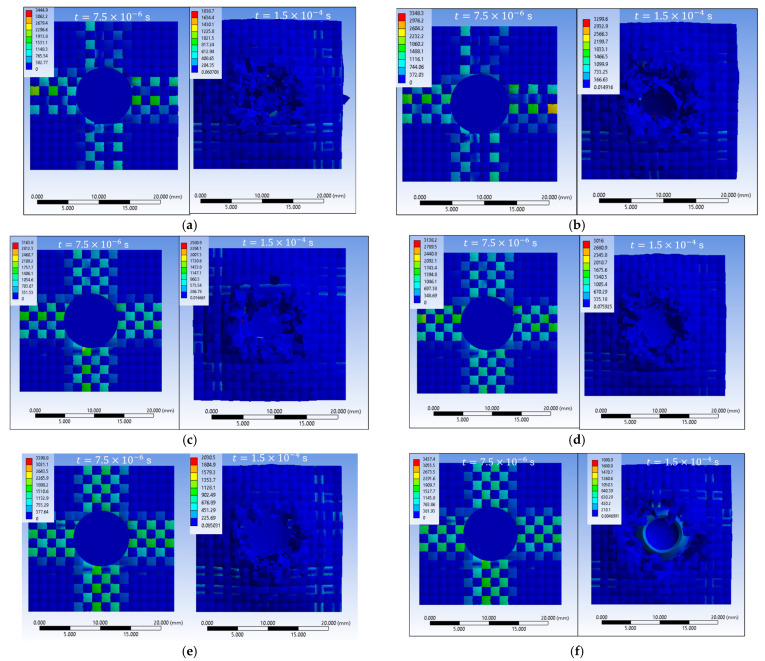

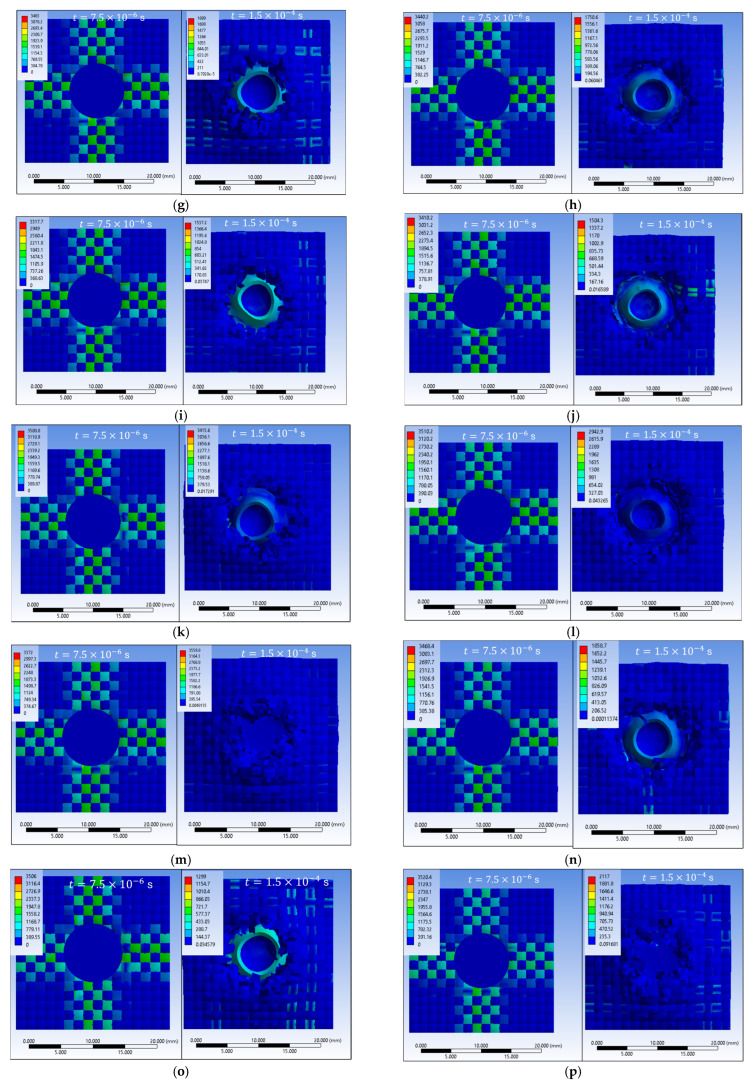
Top view of equivalent stress distribution in the fabric: first and last impact moments (**a**) S1; (**b**) S2; (**c**) S3; (**d**) S4; (**e**) S5; (**f**) S6; (**g**) S7; (**h**) S8; (**i**) S9; (**j**) S10; (**k**) S11; (**l**) S12; (**m**) S13; (**n**) S14; (**o**) S15; (**p**) S16.

The results of the sixteen numerical simulations show that, regardless of the specific scenario, the main wires aligned with the loading direction play the dominant role in stress transfer and energy absorption, while the secondary wires remain weakly stressed during the early impact stage (Figure 4).

At the initial moment of impact (t = 7.5 × 10^−6^ s), all scenarios exhibit high von Mises stress concentrations on the main wires in the contact area, with stress waves propagating either longitudinally or transversely along these wires. The maximum stress values generally lie in the range of 3300–3500 MPa, reaching or exceeding the wire breaking threshold in several cases. This behavior confirms that the main wires are the first structural elements to carry the impact load, whereas the secondary wires contribute marginally at this stage.

In the final stage of impact (t = 1.5 × 10^−4^ s), two distinct response regimes are observed. In scenarios leading to complete perforation, the impactor destroys the fabric along its trajectory, with extensive wire breakage, fragmentation, and loss of structural integrity localized in the impact zone. In contrast, scenarios associated with partial penetration or impactor arrest show a marked reduction in the maximum stress level (down to approximately 1300–2100 MPa), with damage confined to the upper layers and several lower layers remaining intact. In these cases, the impact energy is dissipated through combined mechanisms of compression, bending, lateral wire displacement, and local rupture.

Overall, the comparative analysis demonstrates that stress redistribution along the main wires, together with frictional effects and contact conditions, governs the penetration outcome. High initial stress localization on the main wires initiates damage, while the subsequent redistribution and attenuation of stresses control whether the response evolves toward complete perforation or effective impactor arrest.

**Figure 5:**
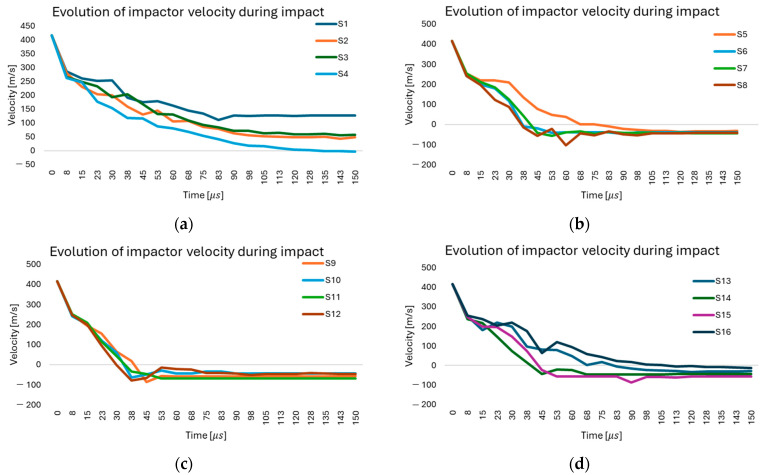
Impactor velocity evolution during impact for modeled scenarios, illustrating dependence on parameters: (**a**) Simulation scenarios S1–S4; (**b**) Simulation scenarios S5–S8; (**c**) Simulation scenarios S9–S12; (**d**) Simulation scenarios S13–S16.

Figure 5 shows the variation in impactor velocity over time for all simulated scenarios (S1–S16). In cases S1–S4 (Figure 5a), where the friction coefficient ranges from 0.0 to 0.1, it is observed that, in scenario S1, the impactor retains a significant residual velocity, which indicates an almost complete penetration of the panel. Increasing the friction coefficient (S2–S4) results in a faster decrease in impactor velocity and, consequently, in a more efficient dissipation of impact energy within the fabric. For scenarios S5–S8 (Figure 5b), with a friction coefficient of 0.2 and 0.3, a more pronounced reduction in velocity is observed, and the impactor is completely arrested within shorter time intervals. The oscillations observed after stopping are attributed to local interactions between yarns and to rebound effects associated with material fragmentation. Scenarios S9–S12 (Figure 5c), with friction coefficients of 0.4 and 0.5, demonstrate that complete impactor arrest occurs in all cases, with differences associated with the energy dissipation mechanisms. At higher friction coefficients, impactor stoppage occurs more abruptly; however, higher local stresses develop, promoting the formation of concentrated rupture zones. Scenarios S13–S16 (Figure 5d), with identical material properties and varying friction coefficients, confirm the same overall trends: lower friction values permit more pronounced penetration, whereas higher friction coefficients enhance energy dissipation and lead to complete impactor arrest, although more severe local stress concentrations are generated. Overall, a clear correlation is identified between the friction coefficient and the residual velocity of the impactor: increasing friction reduces the penetration depth and promotes more effective absorption of impact energy by the fabric.

**Figure 6:**
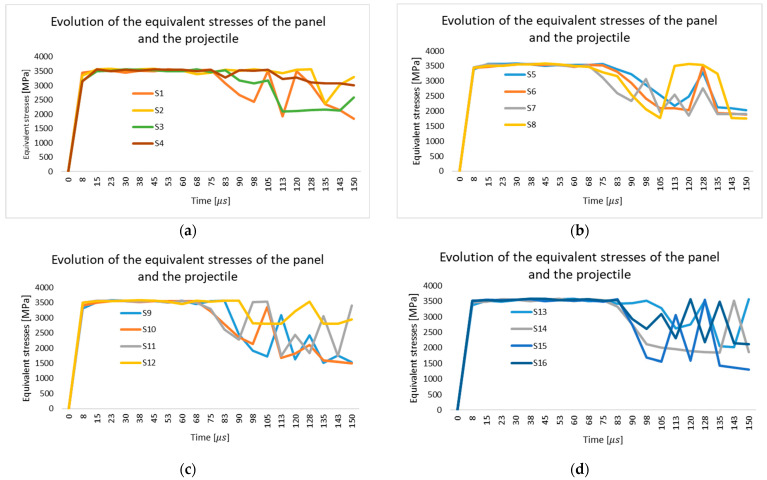
Evolution of Von Mises stress distribution in the panel and impactor during impact: (**a**) Simulation scenarios S1–S4; (**b**) Simulation scenarios S5–S8; (**c**) Simulation scenarios S9–S12; (**d**) Simulation scenarios S13–S16.

Figure 6 illustrates the variation in maximum equivalent stress (von Mises) within the fabric during the impact event for all simulated cases (S1–S16). In cases S1–S4 (Figure 6a), where the friction coefficient ranges between 0.0 and 0.1, the maximum stress is observed to rise rapidly in the first microseconds, reaching values around 3400–3500 MPa, after which stress fluctuations occur. For S1 and S2, a more pronounced decrease is observed in the later stages, indicating yarn rupture and progressive damage localization. In cases S5–S8 (Figure 6b), with friction coefficients of 0.2 and 0.3, the maximum stress is maintained at elevated levels for a longer duration before decreasing, which suggests that the yarns sustain higher loads and contribute more effectively to energy absorption. However, strong oscillations are observed after 80–100 μs, corresponding to yarn fragmentation and redistribution of stresses to adjacent yarns. Cases S9–S12 (Figure 6c), corresponding to friction coefficients of 0.4 and 0.5, exhibit a similar peak stress of approximately 3500 MPa, followed by a sharper decrease after about 70 μs. This behavior indicates that higher friction enhances energy dissipation through frictional contacts, but also promotes more localized rupture, leading to stress drops and oscillatory behavior. Finally, in cases S13–S16 (Figure 6d), identical material properties are retained, and only the friction coefficient is varied. The general tendency remains consistent: higher friction values extend the duration of elevated stress levels, while also generating localized stress concentrations that accelerate yarn rupture, resulting in irregular stress oscillations in the final stage. The comparison of these scenarios confirms that the friction coefficient exerts a strong influence on stress evolution: low friction favors rapid localization and penetration, whereas higher friction enhances load transfer, increases yarn involvement, and improves energy dissipation, albeit at the expense of more fragmented rupture patterns.

**Figure 7:**
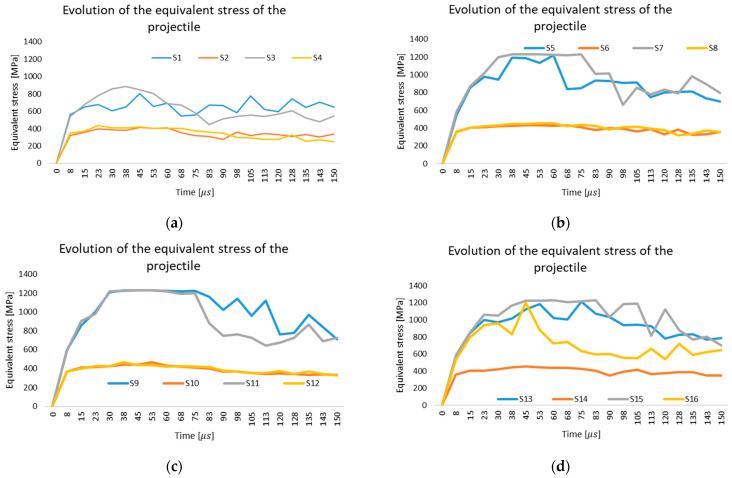
Evolution of Von Mises stress distribution in impactor during impact: (**a**) Simulation scenarios S1–S4; (**b**) Simulation scenarios S5–S8; (**c**) Simulation scenarios S9–S12; (**d**) Simulation scenarios S13–S16.

The von Mises stress histories of the impactor are characterized by a rapid increase immediately after contact, followed by scenario-dependent peaks and oscillations that reflect variations in friction level and impactor constitutive behavior, with higher stresses being associated with increased plastic deformation and a more pronounced interaction with the fabric during penetration or arrest (Figure 7).

**Figure 8:**
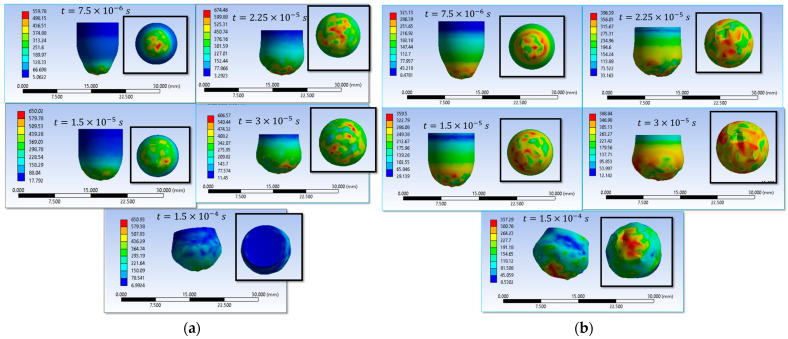
Distribution of von Mises stresses in the impactor at different impact times (**a**) impact scenario S1, variant 1 of the material properties of the impactor (wire-wire friction coefficient is 0.00 and impactor-wire friction coefficient is 0.00); (**b**) impact scenario S2, variant 2 of the material properties of the impactor (wire-wire friction coefficient is 0.00 and impactor-wire friction coefficient is 0.00).

**Figure 9:**
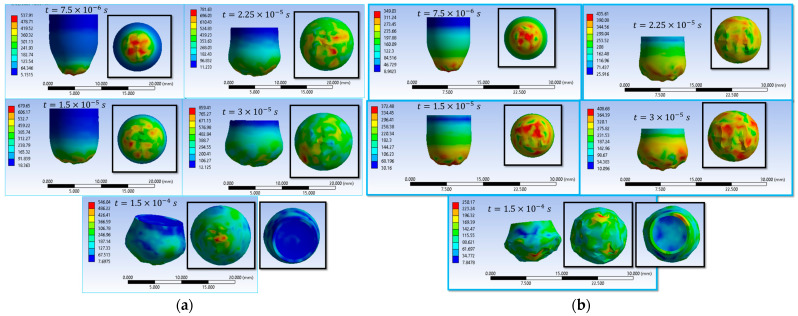
Distribution of von Mises stresses in the impactor at different impact times (**a**) impact scenario S3, variant 1 of the material properties of the impactor (wire-wire friction coefficient is 0.10 and impactor-wire friction coefficient is 0.10); (**b**) impact scenario S4, variant 2 of the material properties of the impactor (wire-wire friction coefficient is 0.10 and impactor-wire friction coefficient is 0.10).

**Figure 10:**
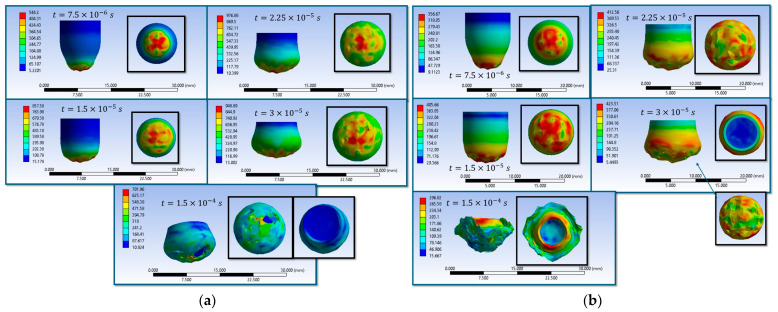
Distribution of von Mises stresses in the impactor at different impact times (**a**) impact scenario S5, variant 1 of the material properties of the impactor (wire-wire friction coefficient is 0.20 and impactor-wire friction coefficient is 0.20); (**b**) impact scenario S6, variant 2 of the material properties of the impactor (wire-wire friction coefficient is 0.20 and impactor-wire friction coefficient is 0.0).

**Figure 11:**
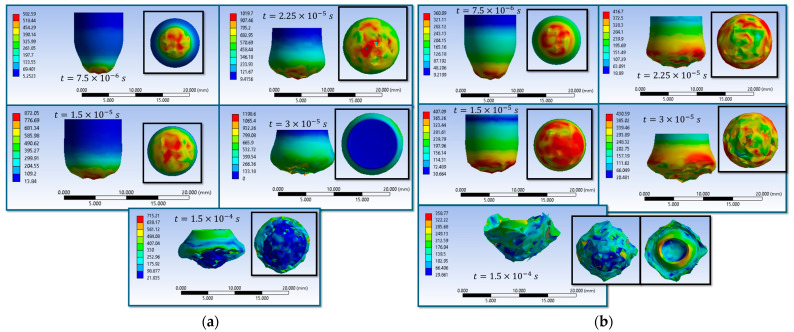
Distribution of von Mises stresses in the impactor at different impact times (**a**) impact scenario S7, variant 1 of the material properties of the impactor (wire-wire friction coefficient is 0.30 and impactor-wire friction coefficient is 0.30); (**b**) impact scenario S8, variant 2 of the material properties of the impactor (wire-wire friction coefficient is 0.30 and impactor-wire friction coefficient is 0.30).

**Figure 12:**
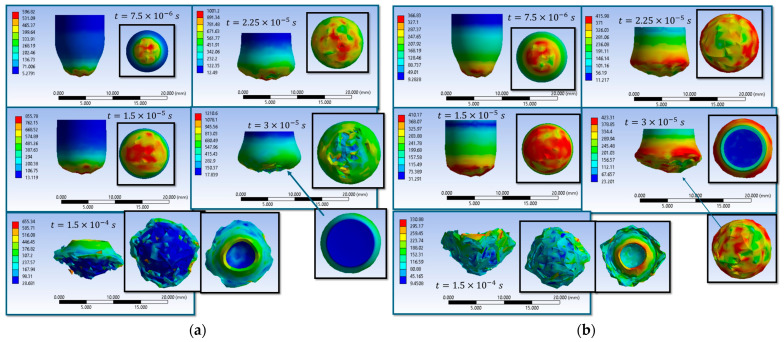
Distribution of von Mises stresses in the impactor at different impact times (**a**) impact scenario S9, variant 1 of the material properties of the impactor (wire-wire friction coefficient is 0.40 and impactor-wire friction coefficient is 0.40); (**b**) impact scenario S10, variant 2 of the material properties of the impactor (wire-wire friction coefficient is 0.40 and impactor-wire friction coefficient is 0.40).

**Figure 13:**
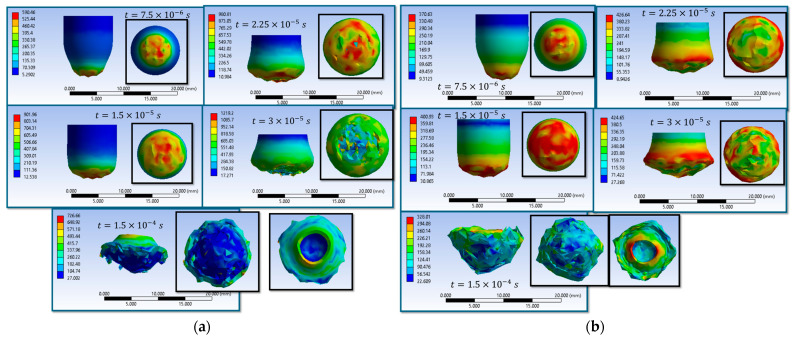
Distribution of von Mises stresses in the impactor at different impact times (**a**) impact scenario S11, variant 1 of the material properties of the impactor (wire-wire friction coefficient is 0.50 and impactor-wire friction coefficient is 0.50); (**b**) impact scenario S12, variant 2 of the material properties of the impactor (wire-wire friction coefficient is 0.50 and impactor-wire friction coefficient is 0.50).

**Figure 14:**
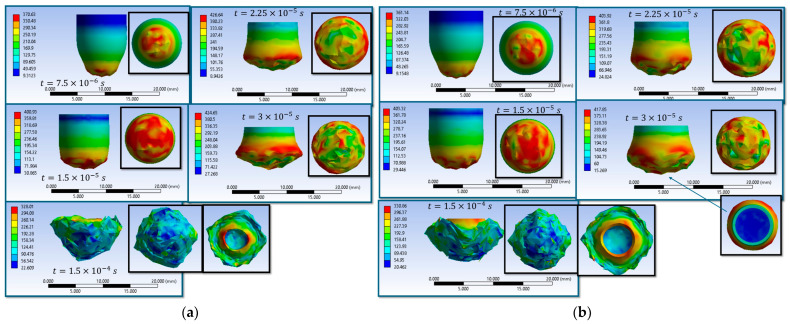
Distribution of von Mises stresses in the impactor at different impact times (**a**) impact scenario S13, variant 1 of the material properties of the impactor (wire-wire friction coefficient is 0.23 and impactor-wire friction coefficient is 0.23); (**b**) impact scenario S14, variant 2 of the material properties of the impactor (wire-wire friction coefficient is 0.23 and impactor-wire friction coefficient is 0.23).

**Figure 15:**
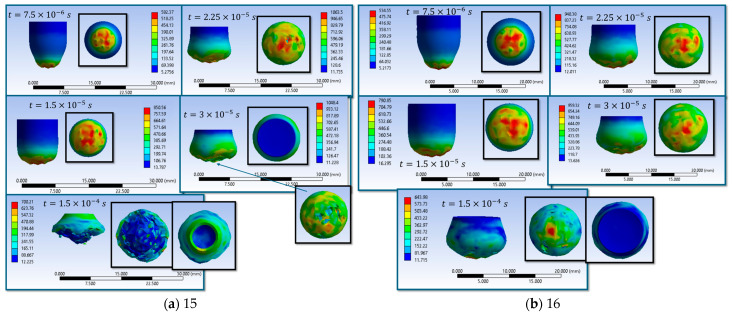
Distribution of von Mises stresses in the impactor at different impact times (**a**) impact scenario S15, variant 1 of the material properties of the impactor (wire-wire friction coefficient is 0.40 and impactor-wire friction coefficient is 0.20); (**b**) impact scenario S16, variant 1 of the material properties of the impactor (wire-wire friction coefficient is 0.18 and impactor-wire friction coefficient is 0.18).

Simulations S1–S16 are used to describe the evolution of the equivalent von Mises stress and the deformation of the impactor during impact with textile panels, thereby highlighting the main mechanisms of energy transfer and dissipation (Figure 8, Figure 9, Figure 10, Figure 11, Figure 12, Figure 13, Figure 14 and Figure 15). In all cases, the initial impact stage ($t=7.5\times 10^{-6}\;s$) is characterized by high stress concentrations at the frontal contact zone, indicating the onset of plastic deformation and the beginning of the mechanical interaction between the impactor and the fabric.

During the intermediate stages $(t=1.5\times 10^{-5}\;\mathrm{and}\;t=3\times 10^{-5}\;s$), stresses are observed to spread from the tip toward the lateral surfaces, and the impactor progressively loses its initial symmetry. Multiple high-stress regions are formed, indicating load redistribution over an increasing contact area. In several scenarios, mushrooming of the impactor, circumferential shear band formation, and asymmetric stress distributions are observed, with these effects being influenced by fabric anisotropy and contact friction conditions. In the most severe cases (e.g., S9, S15, S16), maximum stress exceeds 1000 MPa, revealing critical zones of energy concentration.

In the final stage ($t\;=\;1.5\;\times \;10^{-4}\;s$), the impactor exhibits pronounced global deformation, irregular contours, and a redistribution of residual stresses, which are generally lower than the peak intermediate values. These features indicate substantial energy dissipation through impactor plastic deformation, accompanied by significant damage to the textile layers, including yarn breakage, interlayer sliding, and partial penetration. Overall, the results demonstrate that increased friction coefficients intensify the impactor–fabric interaction, leading to greater deformation and more effective energy absorption, which, in most scenarios, results in arrest of the impactor without complete penetration.

## 4. Model Validation

**Figure 16:**
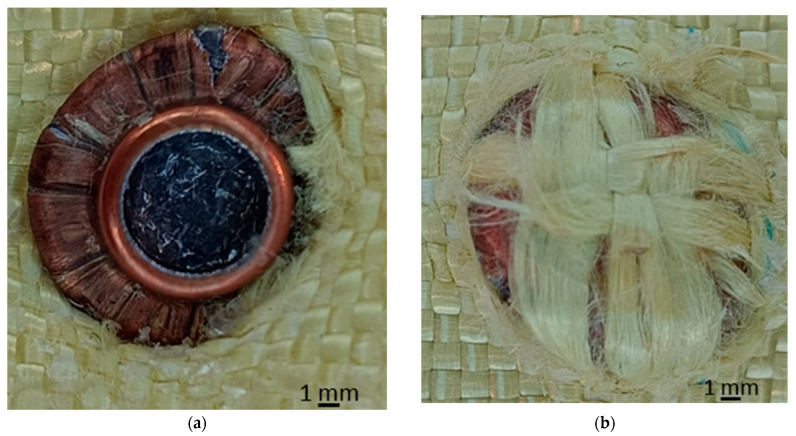
Detailed view of layer 16 of the 24-layer panel impacted by a 9 mm FMJ bullet [48] (**a**) front view; (**b**) back view.

To evaluate the accuracy of the model, the numerical results are compared with experimental observations obtained in [48], where the experimental samples were tested in an experimental testing laboratory following the testing procedure accorded to NIJ 0101.06 (24-layer plain-weave panel, mounted on Roma Plastilina No. 11 and secured with horizontal elastic bands). The experimental case used for validation corresponds to partial penetration, with the impactor being stopped in the panel at layer 16. Figure 16 shows macro details of the impact area: petal formation of the copper jacket and mushrooming of the impactor on the entry face; breaking/removal of the primary yarns and visible pull-out on the upper layers; a circular imprint with lateral displacement of the bundles, consistent with energy dissipation over a moderately extended area.

The numerical results are compared with the corresponding simulations S7 (friction coefficient 0.3, variant 1), S8 (friction coefficient 0.3, variant 2), S9 (friction coefficient 0.4, variant 1) and S10 (friction coefficient 0.4, variant 2), which share the same meso-scale fabric geometry but differ in friction level (moderate to very high) and impactor constitutive formulation. The validation criteria include stopping depth (number of layers traversed until perforation), impactor morphology (degree of mushrooming/petaling of the jacket), width of the affected area, pull-out/flaring pattern in the fabric, and stress distributions and failure sequence (primary vs. secondary yarn breakage).

Major qualitative agreements:

(i) All four scenarios S7–S10, partial penetration is predicted, with the impactor being arrested inside the panel and a circular damage zone dominated by primary yarn breakage and compression of the lower layers, in agreement with Figure 16.
(ii) For scenarios S7 and S9 (variant 1, characterized by a more ductile jacket), mushrooming and petal formation of the jacket are reproduced, together with pronounced lateral yarn pull-out, closely matching the experimental observations.
(iii) The simulated arrest depth is found to lie within the range of 15–17 layers for μ = 0.3–0.4, with a maximum local compaction beneath the impactor tip, which is consistent with the experimentally observed arrest at layer 16.

Of the cases analyzed, the best agreement with the experimental images is obtained for simulation scenario S9 (friction coefficient 0.4, variant 1), for the following reasons: the degree of mushrooming and petaling of the jacket is reproduced most accurately, closely matching the morphology of the recovered impactor; the width of the pull-out strip and the flaring of the bundles around the footprint are consistent with the experimentally observed expansion; the stopping depth is numerically predicted to be within 15–17 layers, in agreement with the experimentally observed arrest at layer 16. Impact scenario S7 (friction coefficient 0.3, variant 1) also shows good qualitative agreement, although slightly smaller differences in lateral expansion and petal amplitude are observed. In contrast, scenarios S8 and S10 (variant 2, stiffer impactor) successfully reproduce impactor arrest but underestimate mushrooming and petal formation, indicating a lower degree of impactor deformation than that documented experimentally.

The agreement obtained for friction coefficients in the range of 0.3–0.4, combined with the ductile constitutive variant of the impactor (variant 1), indicates that moderate-to-high friction at yarn–yarn and yarn–impactor interfaces, together with a metallic jacket capable of petalization, governs both penetration depth and damage morphology. Under these conditions, stress waves are transferred laterally to adjacent yarns with sufficient intensity to arrest the impactor around layer 16, while pronounced primary yarn breakage and pull-out occur in the entry region, in close agreement with experimental observations.

The numerical model captures the dominant physical mechanisms of energy dissipation that are consistently reported in the literature and observed in experimental studies [35,68,69,70,71,72]. In particular, the simulations reproduce:

- Compression of the yarns directly beneath the projectile during the initial stage of impact;
- Propagation of tensile and transverse stress waves along both primary and secondary yarns;
- Progressive yarn stretching, bending, and rupture as the impact evolves;
- Development of localized damage zones and cone-like deformation on the rear layers of the panel;
- Projectile deceleration, arrest, or ricochet depending on the friction conditions and impactor constitutive properties.

## 5. Discussion

Influence of impactor material under frictionless contact (μ = 0).

The comparative analysis of cases S1 and S2, both simulated under frictionless conditions (μ = 0), highlights the decisive role of the impactor’s constitutive behavior in the penetration process. Despite the similar sequence of governing mechanisms (initial compression, tensile wave propagation, and yarn rupture), the two impactor variants exhibit distinct responses in terms of stress distribution and penetration depth.

In case S1, the maximum von Mises stress reaches 2919 MPa at the initial stage and 3519 MPa at t = 1.5 × 10^−5^ s, indicating yarn rupture. At the final stage, stress decreased to 1539 MPa as the impactor is fully arrested, suggesting partial energy absorption through impactor deformation.

In contrast, case S2 records higher initial stresses (3042 MPa) and a comparable peak value (3524 MPa), but the final residual stress remains elevated (1839 MPa), and the penetration depth is more pronounced. This indicates that the stiffer impactor variant transfers a greater fraction of its kinetic energy directly to the fabric, resulting in more localized yarn rupture and increased penetration depth. These findings confirm that, in the absence of frictional dissipation, the impactor’s constitutive response governs the efficiency of energy absorption: ductile impactors tend to absorb energy through internal plastic deformation, whereas stiffer impactors promote stress concentration within the panel, leading to more severe localized damage.

Under conditions where the coefficient of friction between yarns and between the impactor and yarns is μ = 0.1, the differences between scenarios S3 and S4 are attributed solely to the impactor’s material properties. In both cases, the maximum stress values in the fabric reach approximately 3.1–3.5 GPa at intermediate times, exceeding the yarn failure threshold, which results in the rupture of the central yarns and final perforation of the panel. In scenario S3, longitudinal and transverse stress waves are propagated more extensively through the first layers, involving a larger portion of the fabric, while the damage appears more fragmented, with yarns being pushed laterally. By contrast, in scenario S4 the stresses remain more localized beneath the impactor, with pronounced stress concentrators in the contact region, and the damage is concentrated primarily along the axis of impact. Furthermore, the impactor in S3 exhibits more pronounced deformation on the frontal surface and lateral regions, indicating a greater portion of energy dissipation within the impactor itself, whereas in S4 the deformation is less significant, suggesting a stiffer transfer of momentum to the fabric. These results demonstrate that, for μ = 0.1, the constitutive behavior of the impactor becomes the governing factor: a stiffer impactor (S4) promotes stress concentration and penetration along a narrower trajectory, while a more deformable impactor (S3) distributes stresses over a wider area but does not prevent perforation. This confirms that a modest increase in friction is insufficient to arrest penetration, and that either higher friction coefficients or additional structural design strategies are required to enhance load transfer over a broader area.

Under the conditions of μ = 0.2 (yarn–yarn and yarn–impactor), the comparison between scenarios S5 and S6 indicates that a moderate level of friction is sufficient for arresting the impactor within the panel, while the observed differences are primarily attributed to the impactor’s constitutive properties. Both cases initially reach comparable equivalent stress levels (3.40 GPa in S5 versus 3.44 GPa in S6), which exceed the yarn failure threshold; however, their subsequent stress evolution differs significantly. In S5, stress waves are distributed more extensively across the first fabric layers, producing lateral displacement of the yarns and involving a larger portion of the panel in load transfer. In contrast, in S6, the stresses remain more localized beneath the impactor tip, resulting in pronounced compression and bending of the yarns within the contact zone. At the final stage, the residual maximum stress is slightly higher in S5 (2030 MPa) than in S6 (1890 MPa), suggesting that the impactor material in S6 promotes energy transfer over a narrower region, leading to a more localized indentation, whereas the variant in S5 mobilizes a larger fabric volume and dissipates energy over a wider area. Overall, for μ = 0.2, both impactor variants result in complete stoppage, but distinct damage morphologies are observed: S5 is associated with a wider affected zone and lateral yarn displacement, while S6 exhibits a more concentrated central damage region with strongly compressed layers beneath the impactor

A comparison between scenarios S7 and S8, both with the same friction coefficient (μ = 0.3), shows that the observed differences are mainly governed by the impactor’s material properties. In both cases, the equivalent stress rapidly exceeds the yarn failure threshold, leading to localized damage in the central impact region. In S7, the stress remains more concentrated beneath the impactor tip, resulting in partial penetration, with the impactor being retained by the lower layers. Conversely, in S8, the stress distribution is broader, and the damage extends more significantly around the impact area, indicating that the impactor’s material properties promote a more effective transverse transfer of energy. Therefore, while both scenarios exhibit energy dissipation through yarn rupture and compression, S7 is characterized by a localized stress concentration, whereas S8 mobilizes a wider portion of the fabric structure.

The comparison between scenarios S9 and S10, both defined by an identical friction coefficient of μ = 0.4 (wire–wire and wire–impactor), highlights the direct influence of the impactor material properties on the fabric–impactor interaction. In both cases, the maximum equivalent stress values are found to exceed the yarn failure threshold from the early stages, confirming the occurrence of localized yarn rupture in the impact zone. However, the subsequent evolution differs between the two scenarios: in scenario S9, stresses remain more strongly concentrated in the central area, and the impactor tends to penetrate more deeply, with pronounced compression of the yarns in the first layers. In contrast, in scenario S10, the stress distribution becomes more widespread across a larger region, indicating enhanced energy transfer to multiple layers and a more uniform dissipation process. In the final stage, both impactors are fully stopped by the panel; however, in S9 the damage is more localized and severe in the central zone, whereas in S10 the degradation extends laterally but with lower intensity. This contrast clearly demonstrates the role of the impactor material in governing energy transfer mechanisms and the resulting failure mode of the fabric.

Comparing scenarios S11 and S12, for which the friction coefficient is identical (μ = 0.5), the observed differences are primarily attributed to the impactor material properties. In both cases, during the early stages, maximum equivalent stress values exceed approximately 370 MPa and are concentrated at the impactor front. However, in S11 the stresses are more heterogeneous and unevenly distributed, with persistent localized accumulations and more pronounced global deformation observed in the final stage. In contrast, in S12 the stress distribution is more uniform and extends over a wider area, indicating more efficient energy dissipation during the interaction with the fabric. Thus, while both scenarios exhibit significant impactor deformation, S11 is characterized by a more localized and fragile response, whereas S12 suggests a more controlled deformation and a broader redistribution of loads.

Scenarios S13 and S14, where the coefficient of friction is identical (μ = 0.23), highlight the influence of the impactor’s material properties on the impact response. In both scenarios, the initial equivalent stress values exceed the yarn failure threshold (3.37 GPa for S13 and ≈3.46 GPa for S14), confirming the initiation of rupture processes in the first layers. However, the subsequent evolution differs: in S13, stresses are transmitted more uniformly across the impactor surface, leading to a more balanced deformation while reaching higher final peak values (3.55 GPa). In contrast, in S14, stresses remain more localized in the contact zone, resulting in more pronounced global deformation and distinct stress concentrations in specific regions. Therefore, while moderate friction is sufficient to arrest the impactor in both cases, the mechanism of energy redistribution differs significantly. The impactor in S13 engages a larger portion of the panel, leading to more uniform energy dissipation, whereas in S14, energy is concentrated within a narrower zone, producing more severe deformation and a greater loss of the impactor’s initial symmetry.

In this series of scenarios (S1, S3, S5, S7, S9, S11, S13, and S16), the constitutive properties of the yarns and the impactor are kept identical, with the only variable being the friction coefficient applied to the contacts (yarn–yarn and yarn–impactor). Comparative analysis indicates a clear and systematic effect of increasing friction on both the energy dissipation mechanisms and the damage morphology of the fabric. For very low friction coefficients (S1), stresses are rapidly localized beneath the impactor tip, yarn slippage occurs readily, and rupture propagation exhibits a pronounced axial character, favoring penetration and the formation of a strongly damaged central zone. As the friction coefficient increases, a lateral extension of stress waves is observed, together with the engagement of a larger number of layers in load sharing: friction restricts relative yarn slippage, enhances the anchoring of adjacent yarns, and introduces additional energy dissipation at the contacts, generally reducing penetration depth or impactor progression. However, this additional dissipation is not linearly proportional to the friction coefficient. At moderate friction values (e.g., S7, S9), the affected zone becomes wider and the failure more fragmented, whereas at higher values (S11), energy redistribution can generate pronounced local stress concentrations that promote severe localized failures, even when global penetration is reduced. From a practical perspective, increased friction promotes load transfer to adjacent fibers and broadens the damping zone; however, it may also induce localized overstressing, accelerating rupture in critical regions. From the impactor perspective, identical material properties lead to competing effects: at low friction, the impactor advances more easily and forms a penetration channel, whereas at high friction it is more frequently arrested or partially retained, albeit with more dispersed damage and increased fragmentation resulting from yarn rupture.

Overall, the comparison of cases S2 (μ = 0), S4 (μ = 0.1), S6 (μ = 0.2), S8 (μ = 0.3), S10 (μ = 0.4), S12 (μ = 0.5), and S14 (μ = 0.23), performed using the same impactor material variant, clearly demonstrates that increasing friction simultaneously alters both the load transfer mode and the failure mechanisms. A zero friction coefficient (S2) favors unrestricted yarn slippage and strong stress localization along the impact trajectory, leading to pronounced penetration and compression in the central zone. With the introduction of moderate friction (S4–S8), a lateral extension of stress waves is observed, together with the engagement of a larger number of layers in load bearing; under these conditions, the panel tends to retain the impactor through partial penetration or complete stoppage, while damage becomes more dispersed, with yarns being pushed laterally and multiple zones of stress concentration developing. At higher friction levels (S10–S12), frictional contacts further restrict yarn pull-out and enhance inter-yarn anchoring, thereby increasing energy dissipation through friction and reducing impactor progression. However, this effect is not strictly monotonic: large friction coefficients may induce local stress concentrators near the contact region, so that, although the impactor is arrested, regions of high stress and pronounced deformation persist along the trajectory. Case S14 (μ = 0.23) confirms an intermediate response, characterized by a more uniform redistribution of stresses compared to very low friction, without reaching the intensity of stress localization observed at very high friction levels. Overall, for the same impactor material, increased friction enhances stress-wave dissipation and promotes impactor arrest, shifting the response from a penetration-dominated mechanism at low friction toward a more areal response involving multiple layers and widespread damage at moderate-to-high friction, albeit with the potential formation of localized overstressed regions at very high friction values.

The present findings are consistent with those reported in previous studies, which have emphasized that the dominant energy absorption mechanisms in high-performance textile composites include the compression of layers directly beneath the impactor, the stretching of primary yarns, delamination processes, and frictional interactions both between adjacent yarns and at the yarn–impactor interface. These mechanisms are strongly governed by the material properties and the fabric architecture, which collectively control the efficiency of energy dissipation under high-velocity impact conditions [35].

The comparative analysis of the simulation scenarios confirms that the impact response of woven fabrics is governed not only by local fiber properties but also by system-level effects, such as stress-wave propagation and interlayer interactions. These aspects are also emphasized in the classical work of Cunniff, in which scalar criteria were proposed for evaluating impact performance and comparing different fabric configurations [33].

The simulations carried out in this study further confirm the critical role of the friction coefficient in the impact response of woven fabrics. As demonstrated by Duan et al. [72], frictional contacts between yarns and between the impactor and the fabric contribute significantly to energy dissipation by restricting yarn pull-out and promoting load redistribution, which directly influences the governing failure mechanisms and the extent of damage propagation.

The observed variation in impact behavior with respect to the friction coefficient is consistent with the findings reported by Ingle et al. [73], who identified the existence of an optimal range of inter-yarn friction for maximizing energy absorption. Below or above this range, the efficiency of energy dissipation was shown to decrease, either due to excessive yarn slippage or premature locking, leading to more localized failure mechanisms.

In agreement with Zhou et al. [74], enhancing inter-yarn anchoring (“gripping”)—through increased friction or surface treatments—reduces relative slippage and engages a larger number of layers in load sharing, leading to a broader stress distribution and improved energy absorption in woven composite panels.

Consistent with the recent synthesis by Yang et al. [75], both experimental and numerical studies demonstrate that increased inter-yarn friction enhances load transfer to adjacent fibers, broadens the stress distribution zone, and improves the high-velocity impact performance of high-performance textile composites. This aligns with our simulation results, which emphasize the key role of the friction coefficient in energy dissipation and in governing failure mechanisms.

The results indicate that, for the same friction coefficient, the constitutive properties of the impactor significantly influence both its deformation pattern and the damage morphology of the textile panel. This observation aligns with studies on impactor impacts on metallic plates, where it has been shown that the impactor material governs not only its own degree of deformation but also the entry-hole size, the mode of energy transfer, and the distribution of damage within the target [76,77].

## 6. Conclusions

This study was aimed at investigating the impact response of 24-layer plain-weave (1/1) fabrics subjected to high-velocity impact by an impactor described using two distinct constitutive material variants, under different frictional conditions at both yarn–yarn and impactor–yarn interfaces. Numerical simulations were performed in ANSYS Explicit Dynamics to elucidate how friction and impactor material properties govern energy dissipation mechanisms, damage morphology, and the overall deformation resistance of the textile composite panels.

The main findings can be summarized as follows:

➢ At very low friction coefficients (μ = 0), yarns slide freely, and stresses localize strongly along the impactor trajectory, promoting penetration and concentrated failure of the impacted layers.
➢ At moderate friction levels (μ = 0.1–0.3), stress waves spread laterally, involving a greater number of layers in load sharing. In this regime, the impactor is often stopped or partially arrested, with more distributed damage and multiple zones of fragmented yarn rupture.
➢ At higher friction values (μ ≥ 0.4), stronger interfacial anchoring reduces impactor progress but can also create pronounced local stress concentrations, favoring severe localized rupture despite the reduced global penetration.

Impactor material properties are shown to have a direct influence on the interaction mode: a stiffer impactor tends to promote deeper penetration and more concentrated damage, whereas a more ductile variant redistributes a larger fraction of the impact energy through its own plastic deformation, thereby partially limiting penetration and enhancing energy absorption over a wider region of the fabric.

The simulations are validated through comparison with experimental data reported in the literature, which confirms the accuracy and predictive capability of the numerical model. Scenarios such as S9 exhibit the closest agreement with experimental observations, particularly in terms of penetration depth, impactor deformation, and the deformation profile recorded in the viscoelastic clay backing material.

Overall, the study demonstrates that interfacial friction plays a fundamental role in shifting the response from a localized penetration-dominated mechanism to a more distributed energy dissipation process, while the impactor’s material properties govern the efficiency and direction of energy transfer. These findings contribute to an improved understanding of the energy absorption mechanisms in high-performance textile composites and provide valuable guidance for the optimization of composite protective structures through the combined tailoring of fiber properties, interfacial conditions, and impactor characteristics.

## Figures and Tables

**Table 1:** Simulation scenarios: projectile variants and friction coefficients used in numerical analysis.

Simulation Scenario	Notation	Coefficient of Friction	
		Yarn–Yarn	Yarn–Impactor
Scenario 1	S1	0	0
Scenario 2	S2	0	0
Scenario 1	S3	0.1	0.1
Scenario 2	S4	0.1	0.1
Scenario 1	S5	0.2	0.2
Scenario 2	S6	0.2	0.2
Scenario 1	S7	0.3	0.3
Scenario 2	S8	0.3	0.3
Scenario 1	S9	0.4	0.4
Scenario 2	S10	0.4	0.4
Scenario 1	S11	0.5	0.5
Scenario 2	S12	0.5	0.5
Scenario 1	S13	0.23	0.23
Scenario 2	S14	0.23	0.23
Scenario 1	S15	0.4	0.2
Scenario 1	S16	0.18	0.18

Scenario 1 = impactor material variant 1 (JC+EOS). Scenario 2 = impactor material variant 2 (elastic–plastic). Notation S1–S16 corresponds to combined cases of impactor material and friction coefficients applied to yarn–yarn and yarns–impactor contacts.

**Table 2:** Material properties of the yarns (scenario 1 and scenario 2).

Property	Value	Unit
Density	1450	kg/m^3^
Young’s Modulus	83,000	MPa
Poisson’s Ratio	0.35	-
Bulk Modulus	9.2222 × 10^9^	Pa
Shear Modulus	3.0741 × 10^9^	Pa
Yield Strength	3500	MPa
Tangent Modulus	2000	MPa
Plastic Strain Failure (EPS)	0.04	-

**Table 3:** Properties of the impactor jacket material (scenario 1).

Property	Value	Unit
Density	8300	kg/m^3^
Young’s Modulus	1.17 × 10^5^	MPa
Poisson’s Ratio	0.34	-
Bulk Modulus	1.2188 × 10^11^	Pa
Shear Modulus	4.3657 × 10^10^	Pa
Yield Strength	70	MPa
Tangent Modulus	1150	MPa
Specific Heat	385	J/kgK
Plastic Strain Failure (EPS)	1	-

**Table 4:** Properties of the impactor core material (scenario 1 and scenario 2).

Property	Value	Unit
Density	11,340	kg/m^3^
Young’s Modulus	16,000	MPa
Poisson’s Ratio	0.44	-
Bulk Modulus	4.4444 × 10^10^	Pa
Shear Modulus	5.5556 × 10^9^	Pa
Specific Heat	124	J/kgK
Initial Yield Stress	24	MPa
Hardening Constant	300	MPa
Hardening Exponent	1	-
Strain Rate Constant	0.1	-
Thermal Softening Exponent	1	-
Melting Temperature	760	K
Reference Strain Rate	1	1/s
Plastic Strain Failure (EPS)	1	-

**Table 5:** Properties of the impactor jacket material (scenario 2).

Property	Value	Unit
Density	8450	kg/m^3^
Specific Heat	376	J/kgK
Initial Yield Stress	90	MPa
Hardening Constant	282	MPa
Hardening Exponent	0.31	-
Strain Rate Constant	0.025	-
Thermal Softening Exponent	1.09	-
Melting Temperature	1082.8	°C
Reference Strain Rate	1	1/s
Shear Modulus	4.1045 × 10^10^	Pa
Gruneisen Coefficient	2.04	-
Shock EOS Linear, C1	3726	m/s
Shock EOS Linear, S1	1.434	-

## Data Availability

The original contributions presented in this study are included in the article. Further inquiries can be directed to the corresponding author.
